# Microfluidic Organ-on-a-Chip Devices for Liver Disease Modeling In Vitro

**DOI:** 10.3390/mi13030428

**Published:** 2022-03-10

**Authors:** Perizat Kanabekova, Adina Kadyrova, Gulsim Kulsharova

**Affiliations:** 1 School of Engineering and Digital Sciences, Nazarbayev University, Nur-Sultan 010000, Kazakhstan; perizat.kanabekova@nu.edu.kz; 2 Department of Biological Sciences, School of Sciences and Humanities, Nazarbayev University, Nur-Sultan 010000, Kazakhstan; adina.kadyrova@nu.edu.kz

**Keywords:** organ-on-chip, liver-on-chip, liver disease, microfluidic devices

## Abstract

Mortality from liver disease conditions continues to be very high. As liver diseases manifest and progress silently, prompt measures after diagnosis are essential in the treatment of these conditions. Microfluidic organs-on-chip platforms have significant potential for the study of the pathophysiology of liver diseases in vitro. Different liver-on-a-chip microphysiological platforms have been reported to study cell-signaling pathways such as those activating stellate cells within liver diseases. Moreover, the drug efficacy for liver conditions might be evaluated on a cellular metabolic level. Here, we present a comprehensive review of microphysiological platforms used for modelling liver diseases. First, we briefly introduce the concept and importance of organs-on-a-chip in studying liver diseases in vitro, reflecting on existing reviews of healthy liver-on-a-chip platforms. Second, the techniques of cell cultures used in the microfluidic devices, including 2D, 3D, and spheroid cells, are explained. Next, the types of liver diseases (NAFLD, ALD, hepatitis infections, and drug injury) on-chip are explained for a further comprehensive overview of the design and methods of developing liver diseases in vitro. Finally, some challenges in design and existing solutions to them are reviewed

## 1. Introduction

Today, the issue of liver disease is very common. Only in 2017, more than 1.5 billion people had some kind of liver condition, and more than half of them were affected by non-alcoholic fatty liver disease (NAFLD) [1]. Every year, more than 2 million people die due to liver disease [2]. The prevalence of death due to cirrhosis has increased from less than 2% to almost 2.5% starting in 1990 [3]. Statistical data show that the number of deaths due to liver disease in Europe and Central Asia increased primarily due to increases in ALD (alcoholic liver disease). In developing countries, the primary cause of liver disease is hepatitis infections, which can be prevented and treated, but in limited availability [3].

It is difficult to overstate the importance of the liver in a human body. The liver is a key organ in maintaining the metabolism and digestion, immunity, and detoxification of the organism. The liver cells produce substances that are significant for its function. For example, the bile salts, which are produced by liver cells (hepatocytes), emulsify the fats, therefore, facilitating its digestion [4]. Moreover, the liver is responsible for the synthesis of the majority of plasma proteins such as globulins, which are part of the immune system, and coagulation factors, which are involved in blood clotting. Next, the liver is responsible for drug metabolism. Hepatocytes modify the compounds with the CYP450 group of enzymes and then bind to carrier molecules so that they can be excreted in blood or bile for further removal through kidneys and intestine [5]. Although the liver has the essential capacity to regenerate, those functions can be affected by chronic exposure to a range of chemicals such as saturated and trans-fatty acids, alcohol, or chronic inflammation due to viral disease. Such negative exposure to tissue leads to liver diseases.

A common feature of different liver diseases is that the continuous impact of function significantly increases the risks of the replacement of hepatocytes by fibrous tissue.

**Figure 1:**
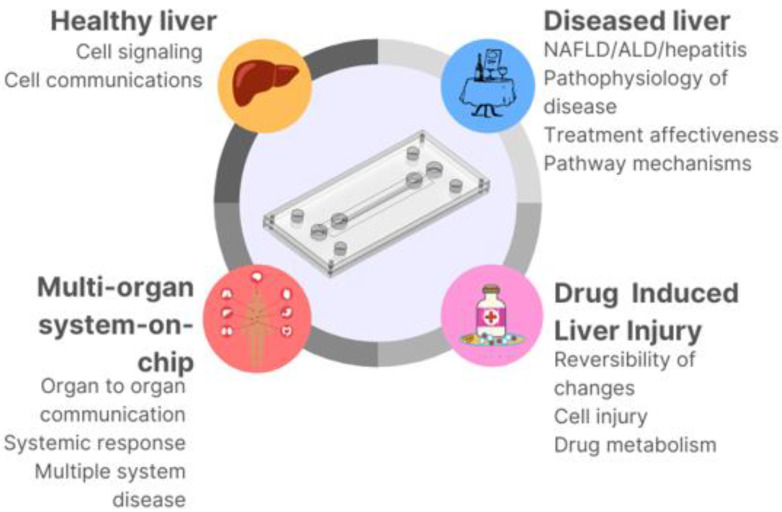
The use of microfluidic devices in different aspects of healthy and liver disease modeling.

In turn, fibrosis and continuous inflammation are essential risk factors for cirrhosis and hepatocellular carcinoma (HCC) as shown in Figure 1 [6]. As the diseases are asymptomatic in the early stages and manifest the symptoms mainly in later stages, timely diagnosis and measures are essential in the course of diseases.

Different in vitro and in vivo models to study the pathophysiology of liver diseases exist. There are several in vitro models-cell culture models, including 2D monoculture and co-culture, 3D cultures, spheroid/organoid cells, human precision-cut liver slices, immortalized cell lines, or sandwich-cultured cell lines [7,8]. Each type of model has its own benefits and limitations. For example, monocultures lack interaction with non-parenchymal cells, limited in control of uniformity in the generation of spheroids and organoids [8]. Moreover, most of those models lack oxygen-induced differences in zonation of hepatic sinusoids [7]. Animal models such as mouse studies allow mimicking liver diseases through diet; for example, a high-fat diet induces NAFLD-like condition or through induction of genetic models [8]. Liver fibrosis induction in mice can also be induced by using hepatotoxins, and hepatitis infection might be initiated by infecting transgenic mice [9]. However, genetic models have different etiology from human NAFLD, making them unfavorable for studying the onset and currency of the condition. Alternatively, rat or minipig models, or primates can be used for studies. The main limitation in terms of non-rodent models is that their metabolic pathways were found to be different, while primate models are much more expensive and raise ethical issues [8].

Emerging technologies and engineering techniques such as microfluidics can facilitate the demand for new in vitro approaches for studying liver diseases. Among such tools, microfluidic organ-on a-chip platforms have seen tremendous growth due to their potential to reduce drug development costs and time. The development of microfluidic devices allows the growth and maintenance of cell lines with the evaluation of function. Biocompatible materials and flow conditions mimic the microphysiological environment, contributing to conducting experiments with different animal cells. The size of microchannels not only reduces the amount of reagent use but also enhances the quick cellular response due to the ability to emulate the microphysiological environment by controlling spatiotemporal parameters such as cell–cell/cell–matrix interaction, shear stresses, excretion of metabolites [10]. Another important aspect of the microsizes of channels is cell-to-liquid ratio, since the volume of the circulating liquid is generally much higher than the volume scale of the tissue is [11]. The composition of the circulating medium can be modified during the flow experiments to achieve more physiological liquid-to-cell ratios [12]. Additionally, drug metabolites can reach higher concentrations due to small volumes of microchannels. Fluidic experiment eluents or medium containing metabolites can be coupled to other devices or microchannels implemented elsewhere on the same platform. Overall, combining microfluidics with tissue engineering can allow achieving even complex architecture of an organ [13,14].

Due to the principal advantages in mimicking microphysiological systems and repeating the microstructure of the human tissue, organs-on-chips have significant potential to replace animal testing models, which are known to exhibit different responses in questions of drug toxicity studies [15]. Moreover, currently drug development relies significantly on animal testing to evaluate the potential drug compound, while organ-on-chip platforms might provide the information based on human cell tests [16]. It opens further potential for personalized medicine, where the cells of patients could be used to access the drug sensitivity in conditions like cancer [17]. These devices are also significant in pathogenesis studies and cell signaling/communication experiments [10].

Organ-on-a-chip technology led to the design of a wide range of tissues on microfluidic devices, including kidneys, heart, and liver [18]. Despite the complexity of biomimicking, the designs of developed devices even allow recapitulating the physiological structures as brain–blood barrier and air–blood barrier in brain and lungs in a wide range of models. For example, the liver-on-chip models range from early models based on monoculture of hepatic cells to more complicated 3D cultures, spheroids, and organoids. The control of fluid flow can potentially allow the development of metabolic zonation [15]. In addition, the possibility to integrate several types of cells with control of biomechanical properties aids in rebuilding the physiological environment of cells [15].

Recently, the exposure of hepatocytes to specific compounds led to the development of disease models on chips. Those are essential for studying the pathological processes at the cellular level in detail. Turning to liver-on-chip devices, the models for conditions like Non-Alcoholic Fatty Liver Disease (NAFLD), Alcoholic Liver Disease (ALD), hepatitis infections, and Drug-Induced-Liver-Injury (DILI) have been developed.

Several reviews have been reported which included some aspects of liver-on-a-chip platforms for liver disease modelling but gave incomplete and brief descriptions of designed diseases on-chips. For example, Hassan et al. reviewed the importance of different cells in the construction of liver-on-chip-platforms and metabolic zonation within the liver [19]. Another review describing the role of non-parenchymal cells in progression indicated the NAFLD as a multi-organ disease in which the functionality of other organs is compromised by the liver state [20]. Similarly, significance of non-parenchymal cells in NAFLD pathophysiology and simulation of different liver compartments was reviewed by Deng, Wei, et al. [21]. The application of liver-on-chip technology to drug discovery was reviewed by Clapp et al., who described the potential use of technology to build models of NAFLD and HBV infections [22]. Concerning the design of microfluidic devices, Moradi with his team presented a detailed review of existing liver-on-chip models, categorizing them by gradient, membrane, or zonation use [23].

Liver diseases can be induced by high drug compounds as well. Microfluidic platforms designed to study the DILI were reviewed by Lin and Khetani, including models involving other organs such as the kidney, nervous system, and skin to evaluate the toxicity of compounds on other organs [24]. The fibrosis modelling using microphysiological platforms was reported by categorizing studies by cell culture techniques such as 2D, 3D models, bioprinted cultures, and describing models by the type of tissue [25]. Van Grunsven et al. presented in vitro fibrosis models, including methods to generate 3D spheroids and microfluidics developed for drug toxicity studies, which can potentially be used for investigating fibrogenesis within the liver [26]. The concept of fibrogenesis in different tissues including lungs, liver, and heart are reported in a review by Hayward et al., as a way for cancer pathogenesis as it is an essential step in the initiation of oncological processes [27].

Here, we present a comprehensive review of microfluidic organs-on-chip platforms used for studying the pathophysiology of different liver diseases from both medical and engineering points of view. The review is aimed at highlighting different perspectives on disease models, methods, and the outcomes of the studies. It also includes other important aspects of organ-on-chip devices such as cell culture models, challenges related to the design of microphysiologic platforms, and the most recent solutions.

## 2. Organ-on-a-Chip Considerations

### 2.1. Cell Culture Models

The scientific world is used to working with regular 2D cell culture for in vitro cell-based experiments. Despite the simplicity of use, those cells cannot mimic the in vivo microenvironment, as a result, the functions and processes within the tissue cannot be fully achieved [28]. To overcome the limitations, different cell culture techniques were developed to better recapitulate the features of the tissue such as the interaction between the cells and metabolic gradients. Table 1 represents the advantages, disadvantages, and current applications of existing cell techniques.

#### 2.1.1. 2D Cell Culture

The cell monolayers are extensively used in studies of drug pharmacokinetics and are essential in the preclinical stage of drug safety/toxicity investigation [33]. Here, the exposure of cells to media and oxygen is homogeneous throughout the surface, and therefore, hypoxic conditions cannot be mimicked. This might be essential in studies of cancer pathophysiology and tissues with metabolic zonation and may affect cell proliferation [34]. On the other hand, 2D cell culture experiments are better in terms of reproducibility, and the cells are easier to handle [25].

#### 2.1.2. 3D Cell Culture

The three-dimensional techniques can be scaffold-based and scaffold-free ones depending on their use of external support [33,35]. Scaffold, designed from natural materials, plays the role of extracellular matrix and aids in cell adhesion, interaction, and exchange of required gases and nutrients. Hydrogels, solid-state scaffolds, might be implemented for the recapitulation of 3D format. Non-scaffold-based cultures include spheroid cultures and organoid cultures [33]. Any type of 3D culture technique allows controlling the concentration gradient of compounds that affect the behavior of the cell such as communication signals, migration to other places, and expression of specific genes [34].

#### 2.1.3. Spheroids

The self-aggregation of the cell using techniques such as hanging drop plates, spheroid microplates, magnetic levitation, microfluidic devices, and bioprinting generates the spheroid structured cells, the shape of which prevents the adhesion to flat surfaces [36]. Those cells as free-floating aggregates, shown in Table 1, were found to better recreate the features of in vivo tissue and excellent simulation of conditions such as low-oxygen state, dormancy, and activation of inhibition of cell death pathway [34]. They can be generated from most cell lines, primary or tumor cells and do not require growth factors or ECM for maintenance [37]. Although spheroids can exist in plates where they were generated, some studies reported that incorporation into scaffolds increases the efficacy in designing tumor models [35].

#### 2.1.4. Organoids

Organoids are closer to their original tissue by functionality and by histological characteristics. In the majority of cases, they are generated from different stem cells and need ECM and growth factors for maintenance [37]. Organoids can be prepared by the same methods as spheroids, but those might lack resembling mechanical forces and physiological cell signals.

To address these issues, microfluidic devices such as organs-on-chips can recreate the microphysiological environment by providing dynamic flow conditions [38]. Organs-on-chips aim to mimic closely the anatomy, physiology, and functionality of a human organ and can be a better model for studying pathophysiology of organ diseases [39].

Table 1 represents the cell culture methods by describing the advantages and disadvantages of using them. It also includes the potential applications of the technique and provides sample images of cell culture grown.

### 2.2. Cell Sources

Physiologically, the liver tissue consists of hepatic cells and non-parenchymal hepatic cells such as stellate cells, Kupffer cells, and endothelial cells. Their role is essential in drug metabolism and disease pathophysiology [40].

There are several cell sources that might be used for in vitro human liver cell culture models. Those are the cell lines, primary hepatocytes (human and animal), and hepatocytes derived from stem cells (induced pluripotent, adult stem cells, and human embryonic stem cells).

Primary human hepatocytes can be ideal for the final drug metabolism testing. While setting up the experiment, they are less likely to be used due to limited availability and inability to proliferate in vitro [40]. In comparison, hepatic cell lines can be propagated and effectively used in experiments, although there is lower expression of enzymes that can be found in primary hepatic cells [41]. The usage of stem cells often raises ethical questions due to sourcing. These cells are in low availability and lack common protocol for use, which makes them more difficult to handle, and they have limited capacity to proliferate.

### 2.3. Dynamic Flow Considerations

Organ-on-a-chip technology is not only based on various cell types or tissue models, but it also heavily incorporates engineering aspects such as spatially guided placement of cells, presence of flow, mechanical stimulation (shear stress), environmental control (O_2_, CO_2_, pH, and nutrients), and integration of sensors [42].

Among them, particularly for liver-on-a-chip platforms, fluid control is of the utmost importance. The fluid control in liver-on-a-chip platforms enables the development of metabolic zonation emulating in vivo conditions of the liver. In in vivo conditions, liver cells are exposed to a gradient of oxygen and hormones [15], and inside the microchannels, this can be emulated by exposure of cells to different axial oxygen gradients [43,44]. The flow rate of the perfusion media varies depending on the research application from 2 nL/min to 5 mL/min [45]. However, the flow rate of a liver-on-a-chip system needs to be chosen very carefully, since shear force caused by flow strongly impacts cells’ intrinsic physiology and hepatocyte’s function overall [46]. Shear rates higher than (5 dyn/cm^2^) 0.5 Pa have been shown to reduce the hepatocyte function [47]. The physiologically relevant range for shear stress inside a single microfluidic device was reported to vary from ~0 to 0.03 dyn/cm^2^ (3 mPa) based on the study done with intestinal epithelial cells [48].

For microfluidic cell culture, oxygen is essential and is mainly transferred using medium flow. Therefore, flow rate contributes not only to the shear stress experienced by perfusion culture, but also is a key element in dissolved oxygen transport to hepatocytes. Previously, flow experiments have been conducted to measure the impact of different medium flow rates ranging from 1 µL/min to 10 µL/min on oxygen consumption of hepatocytes [49]. The study showed that the minimum saturation of cells occurred at 3 µL/min flow rates [49]. Ehrlich et al. reported that an oxygen consumption rate of hepatocytes should be at least 5 nmol/min/10^6^ cells in designing physiological design parameters of liver-on-a-chip platforms in a physiological system suggesting that the device should be able to achieve flow rates higher than 22 µL/min/10^6^ cells [15]. Overall, there should be a balance between a high enough flow rate to ensure sufficient oxygen transport to hepatocytes, but it needs to be balanced with shear stress values.

Dynamic flow culture in organ-on-a-chip platforms also depends on the design of microchannels and cell chambers. Although there are some variations in the designs of fluidic systems for liver-on-a-chip platforms, most of the reported research follow one of the main three designs [15] (1) a simple flat-plate design where cells are cultured on the bottom part of a microchannel [39,47]. (2) A packed-bed design in which hepatocytes aggregates are placed within the 3D chamber [50] and (3) hollow-fiber design in which cells aggregate around fibers delivery oxygen and nutrients [51]. There are several reviews specifically focused on fabrication and design perspectives of organ-on-a-chip devices in the literature [15,52].

## 3. Liver-on-a-Chip Platforms Modeling NAFLD

NAFLD is a chronic condition, which develops due to abnormal lipid accumulation or steatosis, as a result of a fatty diet, lifestyle, and hormonal changes [53]. With progress, the inflammatory component joins the pathological steatotic process, causing steatohepatitis, which consequently leads to fibrogenesis. Histologically, the transition from healthy cells to different degrees of steatosis with subsequent ballooning, fibrosis, and cirrhosis takes place [53]. This condition is asymptomatic in the early stages and might be diagnosed accidentally. The signs and symptoms develop when liver decompensation occurs. As NAFLD is associated with other metabolic conditions such as insulin resistance, obesity, and others, it might manifest and be diagnosed earlier. In addition, the chronic use of drugs such as methotrexate, glucocorticoids, chemotherapeutic drugs, and tetracycline increases the risk of drug-induced hepatic steatosis [53].

**Figure 2:**
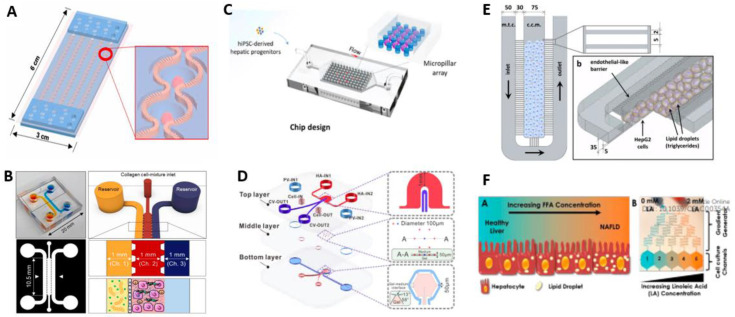
Modeling NAFLD using microfluidic devices. (**A**) Steatochip recapitulating liver microstructure, for growth and differentiation of HepRG cells [54] (reproduced with permission from Teng et al., 2021). (**B**) NASH-on-chip, prepared with hydrogel channel with hepatic, Kupffer and stellate cells [55] (reproduced with permission from Freag et al., 2020). (**C**) NAFLD-on-chip inducing differentiation of hiPSC to organoids followed by steatosis progression [56] (reproduced with permission from Wang et al., 2020 Copyright: ACS Biomater. Sci. Eng. 2020, 6, 5734−5743). (**D**) Schematic illustration of liver lobule chip [57] (reproduced with permission from Du et al., 2021). (**E**) NAFLD on-on-a-chip in 3D and schematic view with HepG2 cell culture [58] (reproduced with permission from Gori et al., 2016; this is an open access article distributed under the terms of the Creative Commons Attribution License). (**F**) NAFLD model on chip generating the gradient of liloleic acid [59] (reproduced with permission from Bulutoglu et al., 2019).

One of the benefits of microfluidic devices is not only the possibility of long-term cell maintenance of 2D and 3D cultures but also that some of them are designed to prepare these cell cultures. For example, the formation of 3D organoids from HepRG progenitor cells was possible by using SteatoChip by Teng et al. (Figure 2A), which represented better recapitulation of liver functions in comparison to conventional 2D cultures [54]. The NASH on-chip, designed by Freag et al., was based on 3D coculture of cells in hydrogel prepared from the collagen, which were studied for biochemical hallmarks of the disease, shown in Figure 2B (2021) [55]. In turn, this validated the significance of 3D structures in the recapitulation of spatial organization to enhance cell communication with controlled conditions. Next, the study by Wang et al. designed the Liver Organoid on Chip, which allowed the formation of 3D aggregates of the liver organoids as well and maintained the long-term perfusion environment as shown in Figure 2C (2020) [56].

In addition to preparing cell cultures and modelling the NAFLD-on-chip devices, microfluidic devices allow the evaluation of drug efficacy on a cellular level. For example, in a study by Gori, Giannitelli hypolipidemic drugs were applied on NAFLD-on-chip hepatic cells, and lipid-lowering activity was confirmed observing lower levels of steatosis [58]. Potentially, the disease-on-chip devices allow not only conducting drug toxicity studies but also drug discovery and drug effectiveness studies as a part of drug approval steps.

The microfluidic devices might be used not only to mimick the microphysiological systems but also to study basic cellular processes in the progression of a condition. For example, Slaughter et al. designed the two-chambered device accommodating liver cells and adipocytes cells (2021) [60]. In this study, the correlation between NAFLD and obesity and diabetes was established by tracking levels of pro-inflammatory cytokines, adipokines, and insulin resistance markers. Although one of the major challenges in the design of liver-on-chip and therefore disease-on-chip platforms is the induction of bile canaliculi, some studies design devices mimic this structure. The NAFLD-on-chip presented in Figure 2D, designed by dual ‘blood’ supply to hepatocytes and recapitulating liver lobule by hexagonally shaped chamber bile canaliculi microstructure development, was successful due to beneficial distribution of hepatocytes and microvessels [57]. Another way to form the 3D cell spheroids was used in a study where AggreWell plates were used to generate cell culture for Steatosis Disease-on-Chip with an optimized ratio of different cells [61]. Moreover, reversal of the pathological process in case of removal of damaging factors was observed, and the effect of anti-steatotic drugs was evaluated. Similarly, Suurmond et al. induced steatosis in spheroid cells generated using AggreWell plates and explored the feedback signaling between them in the pathophysiology of NASH [62]. The three-culture spheroid microphysiological platform was used to induce the NAFLD with progression to NASH by measuring the deposition of the collagen and markers of fibrotic tissue [63]. The increase of inflammatory cytokines and activation of stellate cells were correlated with transcriptomic analysis and gene expression. Moreover, the authors investigated the reversibility of the condition by evaluating the positive effect of treatment drugs in an ongoing study [63].

NAFLD-on-chip models were designed by exposing hepatocyte cells to different fatty acids such as linoleic acid, palmitic acid, or oleic acid for a fixed period. Increased steatosis was observed in cells that were exposed to higher levels of acid. For example, the device which is shown in Figure 2E replicated liver sinusoid by using a system of a grid of microchannels instead of endothelial vascular cells. In this study, treatment of cells with fatty acid in chip conditions resulted in increased cell viability in comparison to static 2D culture, therefore highlighting the benefits of microfluidic devices in animal cell study experiments [58]. Interestingly, one of the studies revealed that the metabolic zonation allowed correlating oxygen concentration to steatosis. Therefore, in the microfluidic device (Figure 2F), the cells with the same concentration of fatty acids, but lower levels of oxygen, had a higher level of steatosis [59].

One of the studies recreated liver acinus with its vascularized form and in coupling with pancreas and adipose tissue from induced pluripotent stem cells [64]. Moreover, the fluorescent protein biosensors were introduced into the device to evaluate the release of reactive oxygen species and insulin resistance. This large-scale study presented metrics for liver-specific biomarkers, which identify the progression from NAFLD to steatohepatitis with the experimental timeline [64].

Overall, microfluidic liver-on-a-chip platforms mimicking NAFLD could be applied to not only to study pathogenesis of disease and drug efficacy but also to emulate other similar steatotic conditions such as metabolic dysfunction-associated fatty liver disease (MAFLD). However, a potential challenge in modeling this condition may be the difficulty in recapitulating systemic diseases such as obesity, diabetes, and hyperlipidemia, which are the diagnostic criteria for MAFLD.

## 4. Liver-on-a-Chip Platforms Modeling ALD

Ethanol is metabolized by alcohol dehydrogenase enzymes present in hepatocytes, resulting in the formation of formaldehyde, which is further metabolized to acetate. Excessive and chronic alcohol consumption leads to the tissue injury associated with ethanol metabolism. One of the earliest reactions of hepatocytes to this type of stress is a fat accumulation of hepatocytes that causes steatosis. It develops in 90% of individuals who consume 4–5 standard doses per day for a long-term perspective (standard dose = 14 g of pure alcohol) and more quickly among binge drinkers [65]. The steatosis progresses to steatohepatitis if lifestyle modifications did not take place with further development of fibrosis and cirrhosis.

Despite the similarities, in NAFLD, the more prominent fatty degeneration of cells can be observed, while in ALD, the inflammatory component is more significant. Moreover, perivascular changes are more frequent in ALD [66]. Although alcoholic drinks have different concentrations of ethanol, it is the effect of ethanol on cells which causes health consequences on liver tissue.

**Figure 3:**
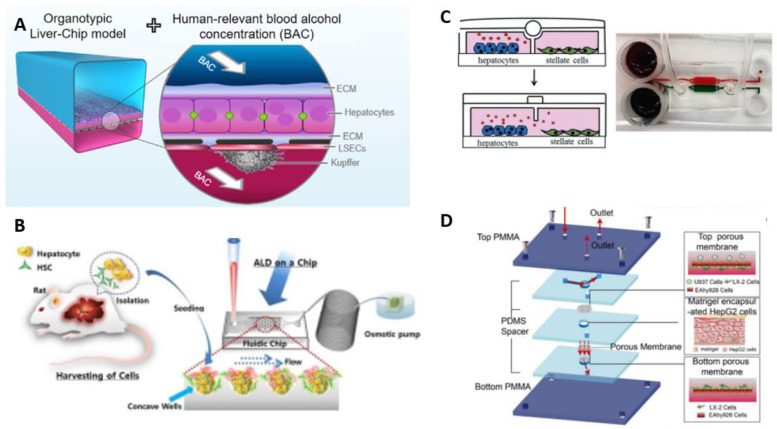
Modeling Alcoholic Liver Disease using microfluidic devices. (**A**) ALD Liver-Chip model with approach of exposing to alcohol [67] (reproduced with permission from Nawroth et al., 2020 Copyright: Cell Reports 36, 109393, July 20, 2021). (**B**) ALD model prepared by culturing primary hepatocytes with stellate cells in PDMS based Liver-on-Chip [68] (reproduced with permission from Lee et al., 2016). (**C**) Liver injury-on-a-chip representing non-mixing channels for co-culture growth [69], adapted from Zhou et al., 2015. (**D**) Liver-chip-based ALD design based on PMMA device with PDMS spaces and PC membrane to maintain cell lines [70] (reproduced with permissions from Deng et al., 2019).

According to many scholars, one of the early indicators of liver function decrease is an alteration of bile canaliculi. In a study by Nawroth et al., the ECM scaffolding was conducted to improve the integrity of bile canaliculi structure, which further allowed to track alteration in canaliculi network in response to alcohol exposure in microfluidic devices as shown in Figure 3A (2021) [67]. As a result, in response to alcohol, the expansion and decreased branching of bile canaliculi was observed, which goes along with the currency of ALD in humans with signs of cholestasis or decreased bile flow.

In addition to an increased inflammatory response in cells to alcohol, the study with the microfluidic device shown in Figure 2B revealed that the higher accumulation of collagen can be observed in liver-on-chip samples with co-culture in comparison to monocultures [68]. This proves the involvement of stellate and Kupffer cells and induction of fibrosis of the tissue in the pathophysiology of liver diseases.

The more complicated microfluidic device, which can be reassembled, was designed by Zhou et al. and included a TGF-β aptasensor to track its secretion from stellate cells and hepatocytes (2015) [69]. The experiment revealed that there is the primary release of cytokine by hepatocyte, which further activates the stellate cells. The release of TGF-β by stellate cells is more significant and mediates the inflammatory component of the ALD. Here the reconfigurable device, represented in Figure 3C, allowed controlling variables in ALD-on-chip such as presence and amount of stellate cells in the co-culture used [69]. Another device designed 3D liver-sinusoid-on-a-chip as shown in Figure 3D to investigate the pathophysiological process of ALD [70]. Here, different cells formed layers recapitulating liver structure and were separated by porous PC membrane to mimic canaliculi and acinus structurally. The damage caused by alcohol was estimated by evaluating DNA damage by reactive oxygen species and established hepatic sinusoid based on results of biomarkers [70].

## 5. Viral Hepatitis Pathophysiology Using Liver-on-a-Chip Devices

There are different viruses, which upon entry into the human body can cause hepatitis infection. Hepatitis A virus (HAV) and Hepatitis E virus (HEV) may enter the organism through consuming contaminated water or food [71]. Hepatitis B, C, and D are transmitted through blood (sharing needles, blood transfusion) and sexual contact. Moreover, hepatitis B infection can be transmitted vertically (from mother to child). While HAV and HEV are not likely to cause chronic infection and treatment is symptomatic, other viral infections are treated by slowing down viral replication only.

The highest probability for HBV to become chronic is during early childhood, although it may happen during adulthood too. In comparison, chronic HCV cases are more common among adults [71]. The injury to hepatocyte cells happens due to immune response to viral particles and infected cells. Chronic inflammation leads to continued hepatocyte death with subsequent liver degeneration. Next, activation of the stellate cells plays a role in fibrogenesis of the tissue, which is followed by cirrhosis and potentially has an increased risk of developing hepatocellular carcinoma [72].

**Figure 4:**
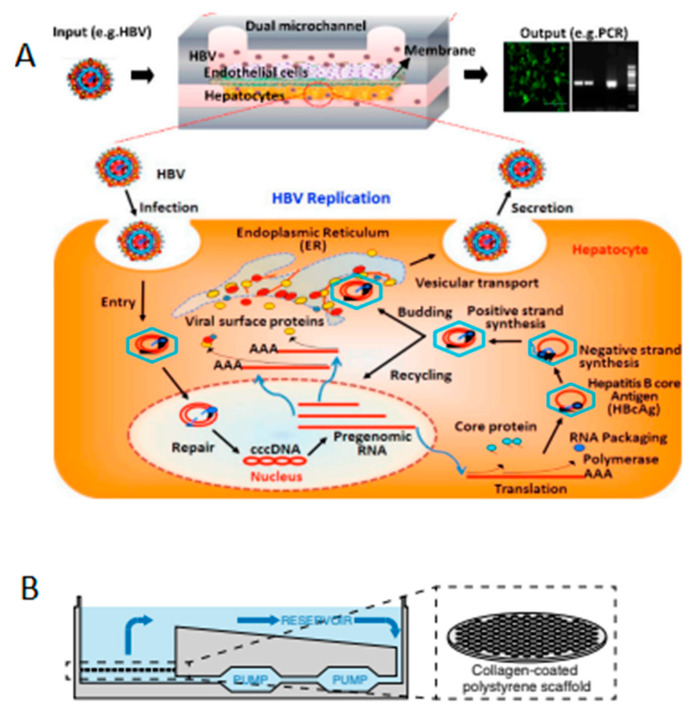
Modeling hepatitis infections using microfluidic devices. (**A**) Human Liver Sinusoid on a Chip design to infect the hepatocytes within the device with HBV virus [74], adapted with permission from Kang et al., 2017. Copyright 2017 by Kang et al.; licensee MDPI, Basel, Switzerland. (**B**) 3D microfluidic liver culture, grown in bioreactor, where circulation is created using pumps [76], adapted with permission from Ortega-Prieto et al., 2018, Copyright © Ortega-Prieto et al., 2018.

Early studies implemented simple microchannel devices to grow cell culture and achieved transfection efficacy sufficient for HBV infection to be higher than in standard tissue culture [73]. Later devices implemented more complex designs with greater number of cells cell types used in studies [73]. For example, the viral replication of HBV in human liver sinusoid on a chip was investigated by detecting viral proteins in the co-culture of primary human hepatocytes and non-parenchymal cells in a study by Kang et al. as shown in Figure 4A (2017) [74]. Here, the cells in the structure maintained viability for almost four weeks, which might potentially be prolonged to study the persistence of viral disease and progression to chronic one. As 3D cultures are known for better recapitulation of the microphysiological environment, one study presented an essential protocol for the preparation of chip and infecting cells with HBV [75]. In this study, the synthesis of cccDNA was achieved indicating the possibility of generating chronic forms of the infection. In another study, the authors investigated the immune response of the cells to the HBV virus and outlined that there was reduced expression of interferons, which are essential for innate immunity [76]. Moreover, the role of Kupffer cells in HBV infection seems to depend on exogenous stimuli of lipopolysaccharides in generating an early response, indicating that the disease is not only affecting the liver tissue but also other organs, primarily the lymphatic system.

## 6. Liver Drug-Induced Injury (DILI) Using Liver-on-Chip Devices

The majority of compounds ingested and absorbed in intestine such as alcohol and drugs are carried by the blood to the liver, making it susceptible to primary injuries by those compounds [77]. Hepatotoxicity was described for a range of compounds, including those that were initially considered to be harmless such as dietary supplements, vitamins, and products such as beans. The biotransformation of compounds can be explained by the alteration of the properties of the compound to activate the drug or to metabolize it for further excretion. However, this can generate byproducts such as reactive oxygen species, which might damage the liver tissue.

Liver fibrosis is a response of the tissue to continuous injury. The external stimuli transform the stellate cells into myofibroblasts, which in turn activate the cascade of changes in the structure of the tissue. Some of those processes are degradation of the external matrix, recruiting other fibroblast-like cells from organisms by chemotaxis, fibrogenesis, and others [78]. The risk factors for this condition are NAFLD, ALD, and hepatic viral infections as described above as well as a range of genetic diseases, multifactor diseases as diabetes mellitus, liver transplantation, autoimmune hepatitis, primary biliary cholangitis, and others [79].

Several liver microfluidic platforms were designed to recreate the DILI in in vitro conditions. For example, one study presented a 3D co-culture device to investigate the liver injury due to acetaminophen with supplementary drugs such as rifampin, omeprazole and ciprofloxacin by measuring the release of lactate dehydrogenase by cells as well as urea, albumin synthesis, CYP, and UGT enzyme activities [80]. The time- and dose-dependent effect of acetaminophen on 3D liver organoids from induced pluripotent stem cells was also studied by Wang et al., where cell injury was evaluated by cell viability assay and functionality of CYP family of enzymes [81]. A more complicated 3D liver sinusoid-on-chip was designed using porous membranes mimicking the environment around sinuses to evaluate the toxicity of acetaminophen in comparison to 2D culture [82]. The results of the study confirmed previous studies in that 3D cultures are more effective in the recapitulation of the liver sinus microenvironment.

To investigate the mechanism and process of DILI, some studies conducted experiments where the ongoing evaluation of liver cells took place. In a study by Bavli et al., the mitochondrial dysfunction in a microphysiological platform was investigated (2016) [83]. The authors used oxygen, lactate, and glucose sensors and recorded a rapid decrease in the oxygen uptake by cells with a subsequent decrease in glucose uptake and lactate production in response to rotenone, with dose-dependent changes for other drugs. Another study investigated drug-to-drug interactions in 3D biomimetic microtissue to evaluate CYP enzyme family induction and inhibition and cell functionality in the chip.

As the hepatotoxic compounds may affect other organs in similar or different ways, some microphysiological platforms involve additional systems. For example, the 3D liver/lung-on-chip platform maintained for four weeks was exposed to aflatoxin B1 [84]. During the experiment, the cilia beating frequency of the bronchial culture and its viability were evaluated along with measuring the activity of liver cell metabolism and injury. Similarly, the effect of inhaled aflatoxin B1 on lung spheroid cells in the presence of liver spheroids was found to be decreased due to the metabolizing effect of the CYP450 enzyme [85]. Another stem cell-derived 3D multi-organoid chip involved the heart, lung, nerves, colon, testis, and blood vessels by creating chips for every tissue with common circulating media [86]. The effect of 10 different drugs, including compounds like vitamin C and ibuprofen, was evaluated on spheroids by measuring specific cell biomarkers at different time points of the experiment. Moreover, the toxicity of drugs such as chemotherapy prodrugs was estimated in the presence and absence of liver organoids. This goes along with existing literature on ability of the liver cells to metabolize the drug, causing the downstream toxicity effect on cardiac tissues [86].

To study the pathophysiology of the fibrosis, Jang et al. used methotrexate to treat 3D layer-membrane co-culture in liver-on-chip [87]. Here, activated stellate cells resulted in an increase in the release of interferon-ˠ, which has a strong association with inflammatory processes and fibrosis. In designing the fibrotic model, the improved cell delivery of non-parenchymal cells in devices with multilayer construction was observed when cells were encapsulated in gelatin bioink during cell printing [88].

## 7. Challenges in Designing Liver Disease-on-Chip Models

Microfluidic devices allow for a wide range of applications and liver-on-chip platforms reported by many scholars reveal functional units of the organ. This allows the recreation of liver parts for detailed study of the metabolism of healthy cells as well as the effect of drugs on cells. Next, it provides the base for the design of disease models such as NAFLD, ALD, or viral hepatitis infections.

It is clear that the creation of disease models is a developing perspective of organ-on-chip devices, as the field still has to address numerous challenges in creating a healthy liver model. Some of these challenges are related to the design of microphysiological structures such as bile canaliculi, and others are related to maintaining and handling cell cultures. However, some of those challenges were solved by simple additional devices or updates in existing ones. For example, the recreation of bile canaliculi and the creation of a stable and long-term 3D culture with immune Kupffer cells or stellate cells in designing healthy liver-on-chip is quite difficult. Mainly, this is due to the fact that bile cells have specific polarity with expression of transport proteins on lumen size, while Kuppffer and stellate cells cannot be randomly introduced within the device, requiring equal distribution and being located in between the hepatocyte cells. In the generation of 3D culture cells or spheroid cells, one of the essential criteria is equal sizing and variability of cells [15]. As a result, some commercially available plates designed to generate spheroids or required diameter with minimal variability were designed. Moreover, 3D liver tissues can be bioprinted, for example, by NovoGen Bioprinter, which allows the generation of tissue that can be maintained for 28 days. Further, this tissue was effective in the recapitulation of drug-induced liver injury experimentally [89]. Using the same NovoGen Bioprinter, the 3D bioprinted tissue was created with primary human hepatocytes and non-parenchymal cells in the study by Norona with her team [90]. Here, the recapitulation of the features of the native liver was studied by evaluating E-cadherin expression by cells, and the induction of fibrosis was done using methotrexate and thioacetamide.

Apart from using different cell culture techniques, there is a wide range of cell types that can be used in microfluidic devices to mimic liver functions. Each type of cell culture has its own limitations. For example, HepaRG cells are known for lower metabolic activity, which can be enhanced by adding 2% dimethyl sulfoxide. However, in a co-culture, this can result in the proliferation of non-parenchymal hepatic cells [91]. The way to facilitate cellular proliferation was suggested by Zuo et al., who generated HepatoCells from primary human hepatocytes, which allowed higher reproducibility by knocking out specific genes [92]. Besides the proliferative capacity, the scholars experience difficulties in maintaining the spheroids homogenous for a prolonged time as with time they tend to lose their volume and decrease in size [93]. In this study, the authors revealed that flow conditions within the microfluidic device have a positive impact on spheroids, resulting in stable volume and stable excretion of albumin for more than a week in comparison to static culture conditions. However, metabolic activity and function of cells including the production of albumin are reported to vary over time [94,95]. For example, Bhise et al. observed a drop in albumin at day 21 in the 30-day long-term hepatocyte culture in a bioprinted liver-on-a-chip device [94]. Overall, while many reports show that liver-on-chip provides a dynamic flow environment for hepatocytes resulting in higher albumin synthesis and urea excretion (detoxification) compared with static cultures [96], values of albumin secretion are normally significantly lower than in vivo conditions. For example, albumin production for primary cells has been reported to be 20–30 μg/(day·10^6^ cells) [15,90] which is typically much higher than albumin levels reported by cell line based liver-on-a-chip platforms (0.6–80 pg/cell/day for HepG2 cells/spheroids) [94]. Since most of the liver-on-a-chip platforms employ cell lines for reproducibility purposes, obtaining real, in vivo-like metabolic activity and biomarker levels remains to be a challenge for the field overall.

One of the other challenges in designing liver-on-a-chip platforms is drug absorption by the materials used to prepare microfluidic devices. Polydimethylsiloxane (PDMS) is one of the most commonly used materials used in microfluidic devices due to its low-cost, optical transparency, elasticity, and gas permeability [39]. However, its main disadvantage is its ability to nonspecifically bind drug compounds, affecting the concentrations of the drugs to which hepatic cells were exposed, which has been well established in the literature [97]. This challenge of organ-on-a-chip devices is being addressed by use of alternative materials and substitutes for PDMS [98].

Moreover, some scholars report the issues related to unequal oxygen concentration within the organ-on-chip different from metabolic zonation within the in-vivo liver. Within the liver, the inflow to the sinusoid comes from arterial blood (rich in oxygen) and portal vein (rich in nutrients) and goes towards the central vein. This facilitates the metabolism of drugs/chemicals to be biotransformed and maintains the oxygen delivery even to less oxygenated zones, which is essential for the expression of some genes [91]. Earlier, it was suggested to decrease the seeding density of cells in vitro to allow the media to accommodate greater amounts of oxygen [99]. Interestingly, although perfused state suggested oxygen delivery, there were limitations in flow rate and capacity. Hence, the introduction of molecules with high oxygen-carrying capacity, mimicking hemoglobin in humans, was suggested [15]. Next, it implemented the introduction of a range of sensors inside of the chip, comprehending the device overall. For example, Farooqi et al. introduced trans-epithelial electrical resistance (TEER) sensors to follow up on the differentiation of cells within microfluidic devices and the integrity of the epithelial layer [100]. Different types of sensors were effectively introduced by de Hoyos-Vega et al., where enzymatic assays to analyze media from hepatocytes, beads-based immunoassays to evaluate secretion of specific growth factors, and electrochemical biosensor detecting the biomarkers released by hepatocytes were tested in microfluidic liver-on-chip [101].

Another challenge related to organ-on-chip platforms is the inability to replicate communication with other organs due to the fact that physiologically, the organs are connected with a wide range of systems. For example, liver organ itself has a dual nutrient supply: the hepatic artery carries oxygenated blood from heart through the general circulation and hepatic portal vein carries the deoxygenated blood with nutrients absorbed from small intestine. Moreover, the hormonal regulation of gastrointestinal tract is far more complicated, making it difficult to recapitulate the communication with other systems. However, there are already some multisystem organ devices such as Gut-Liver Chip that were designed to mimic the correlation between the gastrointestinal tract and liver [102]. The correlation between liver and gut is essential as the majority of ingested compounds are absorbed by the intestine and carried to the liver by the vascular system. Esch et al. recapitulated the liver-intestine model to track the absorption of carboxylated polystyrene nanoparticles by the gut, which resulted in liver injury through increased levels of transaminases [103]. Similarly, a four-organ-chip mimicking intestine, kidney, skin, and liver was designed by Maschmayer et al., where the liver was represented by 3D spheroids [104]. This chip maintained the microphysiological system for 4 weeks and was characterized to be optimal for administration, distribution, metabolism, and excretion (ADME) of drugs for further studies. The different four-organ systems including the heart, muscle, nervous system, and liver were designed to evaluate the response of systems to doxorubicin, atorvastatin, valproic acid, acetaminophen, and N-acetyl-m-aminophenol [105]. The toxicity of drugs on different systems was evaluated by measuring specific tissue biomarkers such as contractile frequency of the cardiomyocytes, electrophysiological recording of neurons, skeletal muscle viability, and metabolic byproducts of the liver after two-day exposure to the components.

In addition to challenges related to design of the device, there are questions that appear within the work such as the most optimal source of cells, using scaffolds or connecting platforms with each other. Next, scientists work on preparation of media that would be universal for multiple types of cells and mimic the blood [18]. For now, in experiments, scientists mix tissue specific media, which might contain byproducts that are toxic for other organs.

Although there are some challenges related to the design and fabrication of the liver-on-chip and disease design, the microfluidic devices have a great potential in disease modelling, drug development, and safety testing. Moreover, it has a promising future in personalized medicine, where the patient-derived tissue would be placed in microphysiological platforms to evaluate drug sensitivity/toxicity.

One of the beneficial potentials and future perspectives of microfluidic devices was reported by Ewart et al., who analyzed Liver-Chip for its ability to predict DILI [106]. This study reported 80% sensitivity of compound toxicities, which has a significant potential in drug development, especially preclinical stage. Moreover, the economic value model in the pharmaceutical industry for increased precision of preclinical studies estimated approximately USD 3 billion in benefits for Liver-Chip studies and for the use of other organ-chips with the same sensitivity up to USD 24 billion in benefits [106].

## 8. Conclusions

Overall, microfluidic devices have a great potential to replace animal experiments for drug testing and disease modelling. Organ-on-a-chip technology has been drawing attention as a new generation alternative to existing in vitro models for drug candidate screening in drug development [107]. The devices can provide cells with a microenvironment, which resembles the in vivo situation more closely [108]. Currently, organ-on-chip devices offer opportunities such as the co-culture of multiple cells in dynamic conditions with a recreation of organ-to-organ communication and integration of biosensors. However, despite the big progress made in organ-on-a-chip platforms used for emulating healthy and liver diseases on-chip, further studies need to be conducted to address existing challenges. One challenge is in variation and inconsistency between different manufacturing batches and different laboratories that produce liver-on-a-chip models. Use of various cell sources and the resulting discrepancy in metabolic function of cells and biomarker values from in vivo conditions continue to make difficult to reproduce not only liver diseases but also a healthy liver environment “on-chip”. PDMS, which has been used for the majority of reported liver models, is also a limitation, but it is actively being replaced by emerging and alternative material substitutes. Complexity and cost of fabrication of organ-on-a-chip is another aspect of interest for the development of robust and reproducible platforms in the future. Despite the limitations, modeling healthy and liver diseases using microfluidic platforms can be accelerated in the near future due to advances in microfabrication and engineering tools as well as due to raising interest within the research community to organ-on-a-chip technology. Modeling liver diseases using liver-on-a-chip platforms can open doors to coupling the platforms with other organ-emulating systems. Multi-organ systems would allow to conduct cross-organ studies and give a more holistic view on liver diseases overall.

## Figures and Tables

**Table 1:** Cell culture methods.

In Vitro Cell Culturing Models	Advantages	Limitations	Applications
2-D (monolayer cultures)	• Easy to grow Low Cost Accessibility [23]	• Loss of tissue specific functions; no cell signaling limited cell–cell/cell–matrix interactions disturbed cellular, mechanical, chemical morphology Uncontrolled access to oxygen and other compounds Viable for limited amount of time [23]	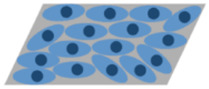 2D culture
3-D (organoids, multicellular spheroids)	• Cell–cell communication Physiological relevance Response to stimuli Cell polarity and phenotype Ability to differentiate, proliferate Tissue viability Drug metabolism [29]	• Cell maturation problems Static: Irrelevant to cell migration and transport Loss of cell shape Hard to collect cellular components for biochemical and genetical analysis No shear stress Necrotic core formation Uncontrolled oxygen and media gradient [30]	Organ Development Tissue Morphogenesis Drug Toxicity tests Creation of cancer organoids-customizing individual cancer treatments Study effect of pathogens [31] 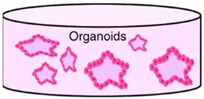 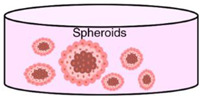 Organoids and spheroids, adapted from Velasco et al. [32] under the terms of the creative commons attribution license
Organ-on-a-chip (microfluidic devices)	• Flow mimicking cell conditions Chemical gradients Easily manipulated cell conditions Relevant amount of shear stress Tissue specific relevant response Recruitment of immune cells Relevant mechanical microenvironment [30]	• High cost Complexity in manufacturing chips More difficult to culture cells inside, required perfusion system The materials of the chip non-specifically absorb drugs [21]	Analysis Recreation interfaces of cellular tissues Study for disease mechanisms Drug toxicity tests/development Could be used in personalized medicine 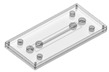 Microfluidic chip

## Data Availability

Not applicable.
